# Acupuncture for chronic nonpulsatile tinnitus: A randomized clinical trial

**DOI:** 10.22088/cjim.9.1.38

**Published:** 2018

**Authors:** Bahram Naderinabi, Soheil Soltanipour, Shadman Nemati, Alia Saberi, Sepideh Parastesh

**Affiliations:** 1 Department of Anesthesiology. Anesthesiology Research Center, Guilan University of Medical Sciences, Rasht, Iran; 2 Department of Community Medicine, Faculty of Medicine, Guilan University of Medical Sciences, Rasht, Iran; 3 Rhino-sinus Research Center, Gilan University of Medical Sciences, Rasht, Iran; 4 Neurosciences Research Center, Neurology Department, Poursina Hospital, School of Medicine, Guilan University of Medical Sciences, Rasht, Iran; 5 Faculty of Medicine, Guilan University of Medical Sciences, Rasht, Iran

**Keywords:** Tinnitus, Acupuncture, Severity, Loudness

## Abstract

**Background:**

There is challenge to find an effective treatment for tinnitus. Few studies were done on the effects of acupuncture on tinnitus. This study evaluated the effect of acupuncture on chronic non-pulsatile tinnitus.

**Methods:**

This randomized double-blind clinical trial was conducted *from December 2014 to September 2015.* Patients suffering from chronic non-pulsatile tinnitus were randomly allocated into two groups: acupuncture *vs.* placebo. They were treated in 15 sessions and at the end of the fifteenth sessions and 3 weeks after completion of the treatment, visual analog scale (VAS) for tinnitus loudness and tinnitus severity index (TSI) questionnaires were completed.

**Results:**

The case group included 26 males and 18 females, and in the control group there were 27 males and 17 females: with mean age of 49.11±1.07 and 55.20±8.33 years, respectively (p=0.005). TSI and VAS before treatment were 43.84±2.81 and 9.56±0.43 in cases and 43.52±2.94 and 9.54±0.45 in controls, respectively. Both measures improved after 15 sessions in cases to 24.82±1.04 and 2.88±0.33, and to 33.16±1.24 and 7.86±0.23 in controls. The changes of TSI and VAS were significant in all groups (p<0.001). TSI and VAS in acupuncture group were lower than placebo group in each session (p<0.001), except TSI in the tenth session (p=0.392).

**Conclusions:**

Acupuncture is effective in reducing the loudness and severity of tinnitus and can be a useful treatment for nonpulsatile chronic tinnitus.

Tinnitus, the perception of sound in the ears or head without the presence of an audible external source, is a symptom, rather than a disease entity, that originates internally without an external auditory input (1, 2). It is relatively common in the elderly (3, 4), but the actual reported prevalence varies according to the surveyed region and definition of tinnitus (4). *One third of adults* in different countries at some points of their life have experienced tinnitus (2-5). About *12 million people in the United States suffer from tinnitus (*6*). According* to the National Center for Health Statistics of the United States, about 32% of all US adults report having tinnitus at one time point in their lives and about 6.4% of them characterizes tinnitus as severe (6-7). According to the national statistics in Iran, at least 5.2 million people in Iran suffer from bothersome tinnitus (8). Tinnitus is more common in men and whites than women and blacks, and its prevalence increases with age, which is not related to the exposure to noise (9-12). Tinnitus is also possible in children, but in this group of patients, it is rarely able to detect it (13). Pulsatile tinnitus is often suggestive of a vascular origin and can be subjective or objective. 

Non-pulsatile tinnitus is almost always subjective and is the most common form of tinnitus. In most patients, tinnitus is associated with hearing loss, but it can also occur in people with normal hearing (14,15). Sometimes there is a structural lesion (*e.g.* acoustic neuroma) or pathological condition (such as Meniere's disease and multiple sclerosis) as the cause of tinnitus and hearing loss, but often, tinnitus and hearing loss occur together without structural lesions or other conditions. In such cases, the symptoms are attributed to ear damages caused by noise or ototoxic materials or other agents (16 -18). 

Otoacoustic emmitsion (OAE) can be used to differentiate between cochlear and retrocochlear pathologies (19). On the other hand, almost half of the patients with peripheral vertigo report having tinnitus and other hearing complaints (20). 

Non-pulsatile tinnitus can be differentiated into mild to severe forms. Mild tinnitus is the noise that people hear occasionally or only in quite places, and is not usually annoying;while severe tinnitus is extremely annoying and often disturbs the patients' quality of life and sometimes, in very severe cases, it can lead to suicide (16).

Acupuncture is a traditional Chinese medical treatment which has been used for thousands of years to treat a variety of diseases as well as to relieve pain (21-25). In 1979, the World Health Organization (WHO) approved the use of acupuncture for the treatment of 41 diseases including ear, nose and throat (ENT) and various types of pain (26-29). The diagnosis system of traditional Chinise medicine (TCM) is based on monitoring the Yin/Yang system and five-phase theory.In this system, the symptoms of the disease are individually divided into mutual and contradictory groups. In this classification, there are eight diagnosis patterns that contain four symmetric symptoms pairs (23). 

There is a theory which states that the therapeutic effect may be attained by inserting needles in selected points which are related to specific organs. Animal and human studies have shown that the stimulation of acupuncture points leads to changes and the release of neurotransmitters such as serotonin, oxytocin and endorphin in the central nervous system (CNS). In addition, functional MRI has shown changes in blood flow in the different parts of the brain caused by acupuncture stimulation (9).

As the result of many studies, acupuncture can lead to early comfort, significant improvement in quality of life, less stress and better sleep after noisy and disturbing tinnitus; whereas, another set of studies showed no statistically significant results in terms of annoying noise in treatment group compared with placebo group (30). There is currently no definitive treatment for tinnitus. Most of the tinnitus treatments are for reducing symptoms (31) and the use of acupuncture for a symptom such as tinnitus is similar to the situation of relieving pain (32). According to the mentioned information and the fact that few studies have been done on the effects of acupuncture on tinnitus, to reduce the impact of or completely cure bothersome tinnitus, a clinical trial has been designed to assess the effects of acupuncture on chronic non-pulsatile tinnitus.

## Methods

***Participants:*** *This *study was a randomized double-blind placebo-controlled clinical trial which has been carried out in the otology and acupuncture clinics affiliated to Guilan University of Medical Sciences (GUMS), North of Iran from December 2014 to September 2015.The proposal of this project was approved by the Research Ethics Committee of GUMS with code of IR.GUMS.REC:1930111206 and registered in Iranian Registry of Clinical Trials (IRCT) number of IRCT: 201404191138N11. 

The required sample size was estimated by WINPEPI software (Version 2.57) based on the results of the reference number 9 as a total of 88 subjects (44 in case group and 44 in placebo group). Eligible individuals included 20 to 65 years-old patients with normal hearing or mild hearing loss (SRT<40 dB), suffering from chronic (i.e. at least 6 months) non-pulsatile tinnitus in one or both sides. Non- pulsatile tinnitus was considered for this study, because *pulsatile tinnitus often has a vascular origin* and logically cannot be treated with this method. Initially a full clinical history taking and thorough examination of the ear, temporo-mandibular joint, and cranial nerves were performed by a neuro-otologist to identify and exclude any underlying diseases associated with tinnitus. The exclusion criteria were: having pulsatile tinnitus, Meniere's disease and other types of vertigo, vestibular schwannoma, otosclerosis, conductive hearing loss or impaired impedance audiometry (IA), pregnancy, mental disorders making difficult to respond to a questionnaire.

**Data gathering:** After the patients fulfilled the informed consent, demographic information and initial examinations were recorded, and then, audiometry tests including pure tone audiometry (PTA), impedance audiometry (IA), speech reception threshold and speech discrimination score (SRT and SDS) were done. Then, patients declared their tinnitus loudness on the basis of visual analog scale (VAS) subjectively, on which number 1 indicated the lowest and number10 indicated the highest tinnitus severity conceivable for the patients. Also, the Persian version of TSI (tinnitus severity index) questionnaire was completed for patients. It is a standard questionnaire with 13 items for quality of life in patients suffering from tinnitus (15). Then, the patients were randomly allocated in two groups of acupuncture (cases) and placebo with sham procedure (controls), using randomized fixed quadripartite blocks.

**Figure 1:**
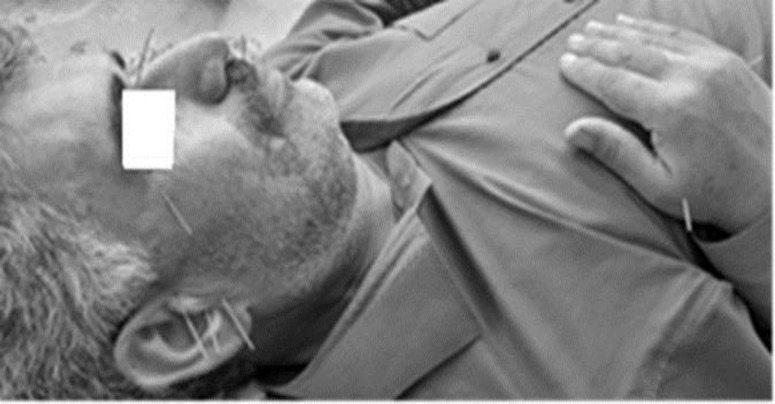
Needle insertion in acupuncture points suitable for treating tinnitus

**Procedure:** Based on previous studies, the acupuncture points suitable for treating tinnitus, including GB2, GB20, SJ21, SI19, SJ17, SJ3, SJ5, LI4, and SI6 points were selected, and the patients received 15 acupuncture sessions (3 times a week). Acupuncture was done by a skilled acupuncturist (B. N.) on specific points. Used needles, filiform or string types, were very delicate and flexible. The size of metal needles was 0.3×0.25 mm. Needles were placed vertically and without anesthesia at the desired points (fig 1). 

In the sham procedure (control) group, the subjects were treated in the points, adjacent to the acupuncture points, but without any effect on tinnitus, using fake needles. These fake needles do not pierce in the skin and are totally pain-free and harmless. The position of the patients and the preparation process were similar in both groups. At the end of the fifth (T1), tenth (T2) and fifteenth (T3) sessions, as well as 3 weeks after the completion of the treatment course (T4), all patients filled the TSI questionnaire and VAS score. During the treatment, patients were examined in terms of potential complications of acupuncture such as infection, - central and peripheral nervous system damage and heart damage, and cases with complications were excluded from the study and managed properly. The data were recorded due to the patients' responses to VAS and TSI questionnaires. 

**Analysis of data: **To test the normality of distribution of the variables, Kolmogrov-Smirnov test was used, and then, the appropriate statistic tests were chosen to compare VAS and TSI from T1 to T4. Mann-Whitney U test and Fisher's exact test or t-test were selected based on the normality of variables. Repeated measurement was applied to determine the trend of changes in the two groups.The values were estimated as mean ± standard deviation (SD). The results were considered statistically significant when the probability of findings occurring by chance was less than 5% (p<0.05). All analyses were performed in SPSS software Version 20 (IBM Company, Chicago). 

## Results

A total of eighty-eight patients participating in this study were assigned with a ratio of 1 to 1 in acupuncture and placebo groups. Their characteristics, after random assignment are summarized in table 1. 

**Table 1:** Demographic characteristics of the study participants

**p.value**	**Placebo group **	**Acupuncture group**	**Variables**	
0.005	55.20±8.33	1.07±49.11	Age (SD±Mean) (year)	
>0.05	27(61.4%)	(59.1%)26	Male	Gender
	17(36.8%)	(40.9%)18	Female	

At the start of the treatment sessions, none of the patients in the acupuncture group were excluded. Three patients in placebo group, two men and one woman with the mean age and standard deviation of 51.00±1.15 years did not complete the study. Thus, analysis was performed on 41 patients from this group. 

At the end of the tenth and the fifteenth sessions, and three weeks after the end of treatment,one patient in the placebo group, four patients in the placebo group, and 12 patients (9 patients in acupuncture group and 3 patients in the placebo group), respectively were excluded from the study. Loss to follow-up in different stages of trial were 2.43% at the end of the tenth session, 9.75% at the end of the fifteenth session and 7.31% in the placebo group three weeks after completion of the treatment. The reason of exclusion was mainly loss of follow-up because of patients' low compliance. Also, 20.45% in acupuncture group three weeks after completion of the treatment have not been followed-up. 

**Descriptive analysis:** The of severity and loudness of tinnitus, based on TSI and VAS respectively, using Kolmogrov-Smirnov test indicated that in the treatment group, tinnitus severity followed a normal distribution, but tinnitus loudness scores did not follow a normal distribution. In the placebo group, none of tinnitus severity and loudness scores followed a normal distribution.Repeated measures ANOVA analysis was used to assess the changes of the severity of tinnitus on the basis of TSI- in case group at the end of the fifth, tenth, and fifteenth sessions, as well as 3 weeks after the end of the course. Mauchly test was used to analyze the sphericity of data, and the result was significant (p<0.001). The Greenhouse-Geisser correction according to the statistics of epsilon=0.5 showed a significant difference between the severity of tinnitus during treatment sessions (F=172.80, p<0.001). 

Post hoc analysis using Bonferroni test indicated that the severity of tinnitus, according to TSI, was reduced significantly in each of the 4 time measure points from the end of the fifth session to 3 weeks after the end of the treatment (p<0.001) (table 2). 

Such analysis was performed in placebo group. The result of Mauchly’s test was significant (p<0.001). A significant difference was between the severity of tinnitus during treatment sessions (epsilon=0.55, F=33.19, p<0.001). The severity of tinnitus, according to TSI, was significantly different between the fifth and tenth (p<0.001) and between the tenth and fifteenth sessions (P=0.008), but there was no any difference between the fifteenth session and 3 weeks after the end of the treatment (table 2).

**Table 2:** Comparing the tinnitus severity index and tinnitus loudness during treatment evaluation sessions in the two study groups

		**Placebo Group**		**Case Group**		
**P** **	**P** *	**Tinnitus Loudness/VAS** **(Mean± SD)**	**Tinnitus Severity Index** **(Mean± SD)**	**Tinnitus Loudness/VAS** **(Mean± SD)**	**Tinnitus Severity Index** **(Mean** **±** ** SD** † **)**	**Treatment evaluation sessions**
>0.05	>0.05	9.54±0.45	43.52±2.94	9.56±0.43	43.84±2.81	Before treatment
<0.0001	0.001	9.18±0.17	37.21±1.35	7.58±0.23	41.40±1.05	The end of the fifth session (T1)
>0.0001	0.392	8.16±0.23	34.29±1.19	5.36±0.24	34.02±1.00	The end of the tenth session (T2)
>0.0001	>0.0001	7.86±0.23	33.16±1.24	2.88±0.33	24.82±1.04	The end of the fifteenth session (T3)
>0.0001	>0.0001	7.81±0.23	33.13±1.29	2.25±0.27	23.11±1.03	3 weeks after the end of the period (T4)
		< 0.001	< 0.001	< 0.001	< 0.001	p***

† :Standard Deviation

P* :Mann-Whitney U test (Tinnitus Severity Index)

P**: Mann-Whitney U test (Tinnitus Loudness)

P***: Repeated Measurement ANOVA

**Figure 2:**
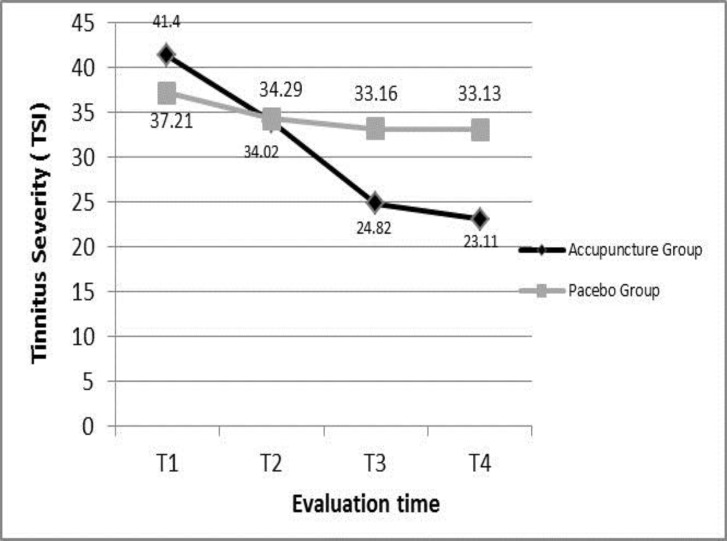
Comparing the mean of tinnitus severity (TSI) between acupuncture and placebo groups in evaluation time points. Covaries appearing in the model are evaluated at the following values. Age= 52.3333.

Figure 2 also illustrates the difference of mean tinnitus severity (TSI) between two groups in 4 evaluation time points and the trend of the changes. In spite of significant difference in term of age between two groups estimated by two independent t-test (p=0.005), covariance analysisshowed that it does not have a role in predictive model of tinnitus severity difference between two groups (P=0.144). As it has been noticed in table 1, TSI in acupuncture group was lower than placebo group in each evaluation sessions (p<0.001) except at the end of the tenth session (p=0.392). To assess the changes of the loudness of tinnitus –on the basis of VAS score- in acupuncture treatment group in different sessions, as well as 3 weeks after the end of the course, the result of Mauchly’s test was significant (p<0.001). Furthermore a significant difference was between the loudness of tinnitus during treatment sessions (epsilon=0.71, F=234.57, p<0.001). 

There, loudness of tinnitus reduced significantly in each of the 4 time measure points (p<0.001) (Table 2). In placebo group at different sessions of follow-up, the result of Mauchly’s test was significant (p<0.001). A significant difference was between the loudness of tinnitus during treatment sessions (epsilon=0.52, F=32.81, p<0.001). The loudness of tinnitus was significantly different between the fifth and tenth sessions (p<0.001). 

There was no significant difference between the tenth and fifteenth sessions and 3 weeks after the end of the treatment (table 2).

T1: fifth session, T2: end of the tenth session and T3: end of the fifteenth session, T4: 3 weeks after the completion of the treatment course

**Figure 3:**
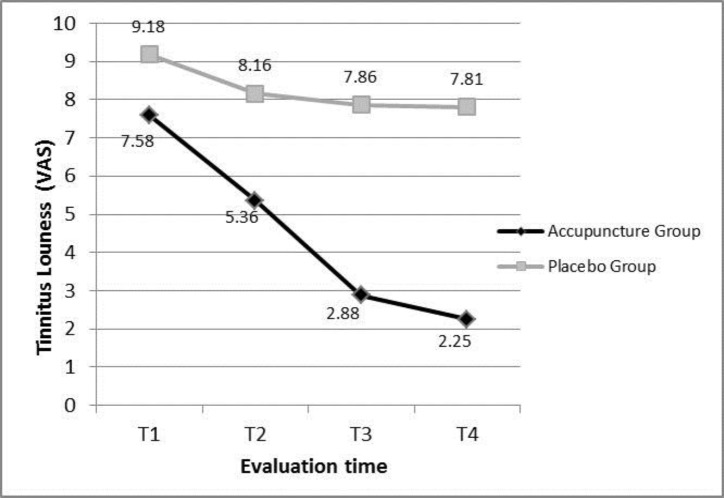
Comparing the mean of tinnitus loudness (VAS) between acupuncture and placebo groups at evaluation time points. Covaries appearing in the model are evaluated at the following values. Age= 52.4658

Figure 3 also illustrates the differencse of mean tinnitus loudness (VAS) between two groups at 4 evaluation time points and the trend of the changes. In spite of significant difference in terms of age between the two groups estimated by two independent t-test (P=0.005), covariance analysis showed that it does not have a role in predictive model of tinnitus loudness difference between the two groups (P=0.095). 

As it has been noticed in table 1, VAS in the acupuncture group was lower than the placebo group in each evaluation session (p<0.001).

T1: fifth session, T2: end of the tenth session and T3: end of the fifteenth session, T4: 3 weeks after the completion of the treatment course

## Discussion

A comparison between acupuncture and placebo groups showed that there were significant differences in tinnitus severity and loudness between both groups at the end of the fifth session, but surprisingly, the difference in TSI was more significant in placebo group. At the end of the tenth session of treatment, a significant difference was found between the two groups only in VAS but not in TSI, but and at the end of the late sessions, the differences by both measures of VAS and TSI were better using acupuncture compared with placebo group. Rogha *et al.* concluded that the mean of both tinnitus severity index and the mean of tinnitus loudness decreased significantly only in the case group after 5 and 10 sessions of treatment, however Rogha *et al. *did not follow-up the patients more than ten sessions of treatment (9) such as in Jeon *et al.’s* study (33). Whereas we confirmed the potential effectiveness of acupuncture on the longer duration of treatment and 3 weeks follow-up, and we observed the same trend in decrease of tinnitus severity in both groups from the tenth to fifteenth sessions, in which this trend was greater in acupuncture group compared with placebo group. Axelsson *et al. *(34) and Vilholm *et al.* (35) who assessed tinnitus severity after 30 and 25 sessions of treatment, found no significant difference in tinnitus parameters after completion of treatment. 

In acupuncture group, the severity of tinnitus according to TSI, was reduced significantly in each of the 4 time measure points. In placebo group, such reduction was also obtained in the treatment course, but not between fifteenth treatment session and 3 weeks after the end of the treatment. Also, in acupuncture group, the loudness of tinnitus, based on VAS, was reduced significantly in each of the 4 time measure points, whereas in placebo group, such reduction was observed only in the early treatment course, and there was no significant difference in other assessment times.

In Jeon *et al.’s *study, the mean percentage of change in VAS 3 months after was higher in the treatment group than the placebo group (33) while in Vilholm et al.’s study, VAS showed no statistical difference between the two groups in a four-month period starting one month before the first treatment until one month after the last treatment (35). In both studies, VAS was not used for scaling the loudess of tinnitus as we utilized it .It was used for quantification of severity and annoying effect of tinnitus. In Axelsson et al.’s study ,no significant difference between acupuncture and placebo was found in annoyance, awareness or loudness of the tinnitus which was measured by VAS (34). In addition, in Okada et al.’s study (32) after 5 sessions of treatment, there were significant differences in the VAS scores which were the same as mentioned above, and the difference was greater in the treatment group.

The present study used VAS to measure loudness and TSI for severityof tinnitus, and the results will be more generalizable. *Rogha et al.* used TSI to measure tinnitus severity such as our study and special tinnitus loudness questionnaire for scaling its loadness (9) that such measurement is recognized in their study and ours from previous studies. In this study, we examined the effects of acupuncture on a larger sample size of patients (n=88), while the other previous studies examined smaller number of patients (9, 32-37).

In the present study, two groups were similar in terms of gender but different in age. In the acupuncture treatment group, 59.1% of patients were men and 40.9% of them were women, and in the placebo group 61.4% of patients were men and 38.6% of them were women. There was no significant difference in sex distribution in two groups, while in Azevedo et al.’s study (36), more than 60% of all patients were women. In Okada et al.’s study (32), 61.8% of all patients were women, while in Axelsson et al.’s study (34), all patients were men, and there was a significant difference in the gender distribution in both groups. Considering the confounding effect of gender on the response to such treatment, our results can be more conclusive.

In spite of significant difference in terms of age between two groups, covariance analysis concluded that it does not have any role in predictive model of tinnitus severity and loudness difference between two groups.

In conclusion acupuncture was more effective in reducing the loudness and severity of tinnitus based on VAS and TSI and can be used as a good treatment option for chronic non-pulsatile tinnitus, considering that no medical treatment has not been yet useful and effective for tinnitus.
